# Muscle-derived extracellular vesicles mediate crosstalk between skeletal muscle and other organs

**DOI:** 10.3389/fphys.2024.1501957

**Published:** 2025-01-08

**Authors:** Jiajie Jia, Lu Wang, Yue Zhou, Peng Zhang, Xiaoping Chen

**Affiliations:** ^1^ National Key Laboratory of Human Factors Engineering, China Astronaut Research and Training Center, Beijing, China; ^2^ Department of Exercise Physiology, Beijing Sport University, Beijing, China; ^3^ National Key Laboratory of Space Medicine, China Astronaut Research and Training Center, Beijing, China

**Keywords:** extracellular vesicles, skeletal muscle, microRNAs, crosstalk, cargo

## Abstract

Skeletal muscle (SKM) has crucial roles in locomotor activity and posture within the body and also functions have been recognized as an actively secretory organ. Numerous bioactive molecules are secreted by SKM and transported by extracellular vesicles (EVs), a novel class of mediators of communication between cells and organs that contain various types of cargo molecules including lipids, proteins and nucleic acids. SKM-derived EVs (SKM-EVs) are intercellular communicators with significant roles in the crosstalk between SKM and other organs. In this review, we briefly describe the biological characteristics, composition, and uptake mechanisms of EVs, particularly exosomes, comprehensively summarize the regulatory effects of SKM-EVs on the function of, which include myogenesis, muscle repair and regeneration, as well as metabolic regulation. Furthermore, we explore the impact of SKM- EVs on various organs including bone, the cardiovascular system, adipose tissue, and nervous system. As emerging evidence suggests that SKM-EVs are involved in the development and regulation of type 2 diabetes (T2D), systemic inflammation, and other chronic diseases, we also highlight the potential of SKM-EVs as therapeutic targets and diagnostic biomarkers, emphasizing the need for further research to elucidate the complex mechanisms underlying intercellular communication in physiological and pathological contexts.

## 1 Introduction

Skeletal muscle (SKM) is a type of striated muscle that is under voluntary control and is responsible for body movement, maintenance of posture, and generation of heat through contraction and relaxation (Mueller-Wohlfahrt et al., 2013). In addition, SKM has also been identified as an actively secretory organ. A variety of bioactive cytokines and factors (myokines) are, synthesized and released by muscle fibers and, participate in a wide range of intercellular communications through endocrine, paracrine or autocrine pathways (Pedersen, 2013; Iizuka et al., 2014). For instance, myostatin, LIF, IL-6 and IL-7 are involved in muscle hypertrophy and myogenesis (Lightfoot and Cooper, 2016; Cirillo et al., 2017), and brain-derived neurotrophic factor (BDNF) and IL-6 participate in AMPK-mediated fat oxidation (Matsumoto et al., 2020; Katashima et al., 2022). These findings enhance our understanding of the importance of SKM in physiological and pathological regulation.

Recently, studies have focused specifically on novel intercellular communicators termed——extracellular vesicles (EVs). EVs are small membrane-enclosed structures that are secreted by cells into the extracellular environment. They play a crucial role in cell-to-cell communication by transporting proteins, lipids, and various types of nucleic acids such as mRNA and microRNA between cells (Yang D. et al., 2020; Yue et al., 2020; Gurunathan et al., 2021).

SKM, as one of the largest organs in human body and accounting for about 35%–40% of an adult’s body weight, is an EVs-rich potential resource pool. Aswad et al. demonstrated that fluorescently labeled skeletal muscle-derived EVs (SKM-EVs) injected into mice were to be taken up by a variety of organs, including the lung, liver, spleen, brain, heart, and pancreas, suggesting that SKM-EVs may mediate crosstalk between SKM and other organs (Aswad et al., 2014). Moreover, levels of circulating EVs and their contents have been found to be altered in muscle disorders and metabolic-related states, such as metabolic dysfunction, sarcopenia, and physical fitness (Aoi and Tanimura, 2021). SKM-EVs have also been reported to actively participate in regulation of the development of type 2 diabetes, systemic inflammation, and other chronic diseases (Donaldson et al., 2013; Barlow and Solomon, 2018; Su et al., 2021; Yamaguchi et al., 2023). These results indicate, a probable role of SKM-EVs in modulation of metabolic processes in various organs.

However, despite the large number of studies that have investigated the roles of SKM-EVs, there has not yet been any systematic and comprehensive of their functions in the interaction between SKM and other organs. In particular, questions remain regarding how SKM-EVs regulate the functions of various organs under different physiological and pathological conditions and the specific mechanism underlying this regulation.

**FIGURE 1:**
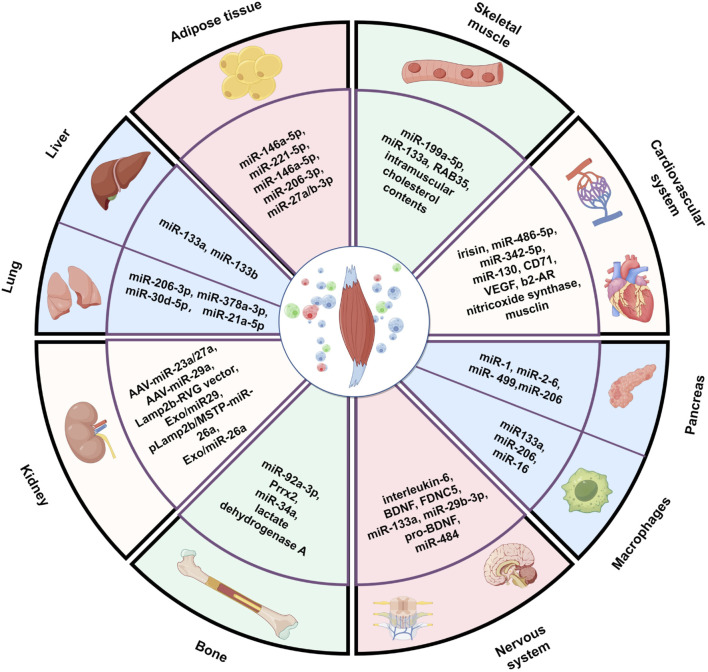
Muscle-derived extracellular vesicles mediate crosstalk between skeletal muscle and other organs. The figure created by Figdraw (accessed on December 13, 2024).

Therefore, in this review, we aimed to comprehensively summarize research progress to date on the crosstalk between SKM and other organs mediated by EVs. We focus on the following aspects: (1) the biological characteristics of SKM-EVs, including their production, composition and uptake mechanism; (2) the regulation by SKM-EVs of the functions and health of SKM itself; and (3) the effects of SKM-EVs on the functions of various other organs (such as the bone, nervous system, cardiovascular system, adipose tissue, and kidney) (Figure 1).

## 2 Overview of extracellular vesicles (EVs)

### 2.1 Classification and characteristics of EVs

**FIGURE 2:**
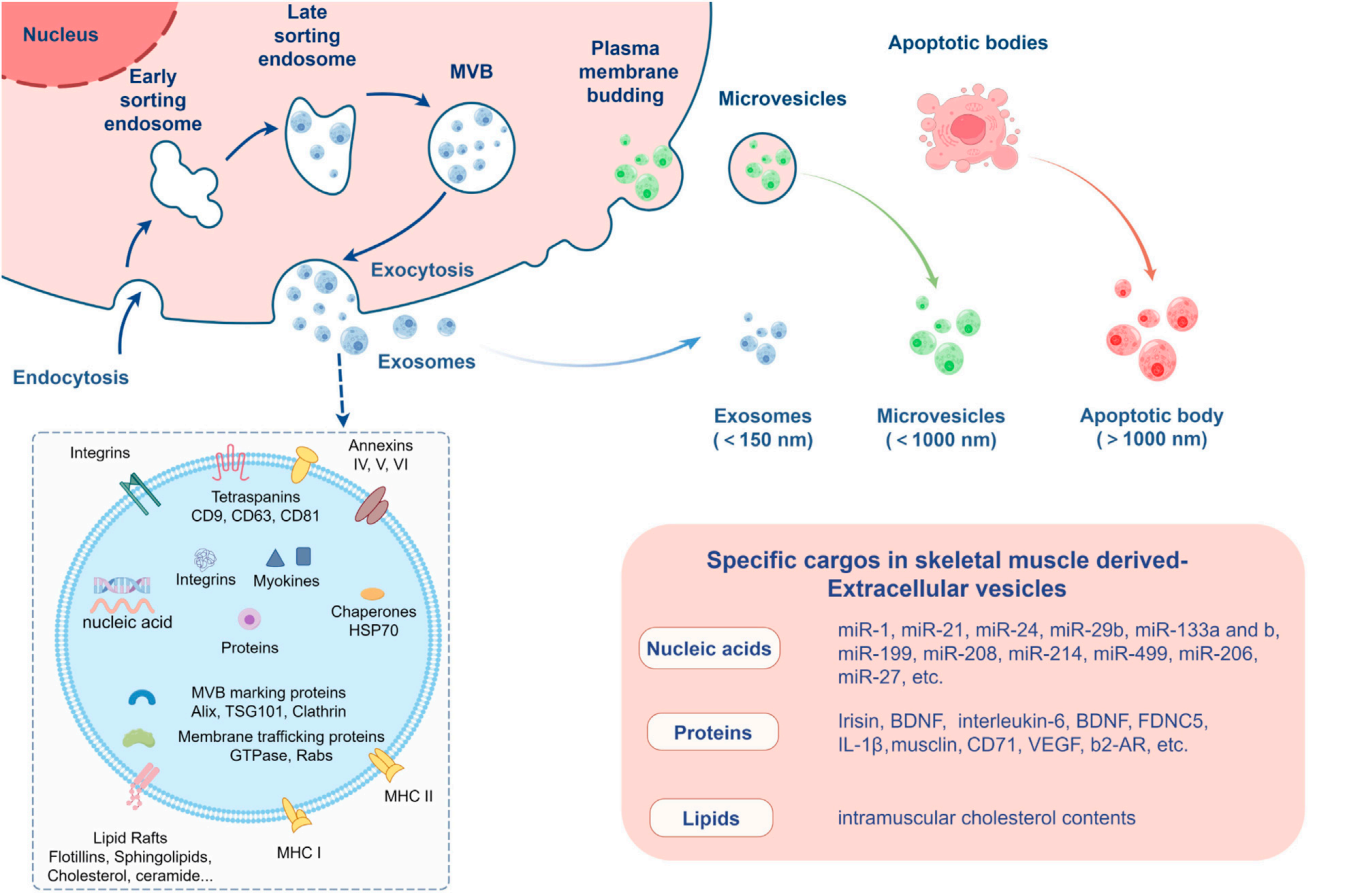
Extracellular vesicles secreted by skeletal muscle. The figure created by Figdraw (accessed on December 13, 2024).

EVs can be classified into different types based on their size and biogenesis, including exosomes, microvesicles, and apoptotic bodies (Figure 2). Exosomes are the smallest type of EVs, typically ranging from 30 to 150 nm in diameter, and are formed through the inward budding of the multivesicular body (MVB) membrane. Exosomes are secreted in various biological fluids including serum, saliva, urine, ascites, and cerebro-spinal fluid (Chung et al., 2019; Gurunathan et al., 2021) and enriched in a variety of bioactive molecules, including proteins, nucleic acids and lipids, and can transfer signals to recipient cells; thus, they also have a critical role in cell-to-cell communication (Wei et al., 2021). Exosomes can be selectively taken up by nearby or distant cells and reprogram recipient cells based on their bioactive compounds they contain. They are ultimately degraded by lysosomes or integrated into the plasma membrane after exerting their effects (He et al., 2018; Van Niel et al., 2018). Microvesicles are organelles that range in size from 100 to 1,000 nm and are secreted by cells through the outward blebbing of the plasma membrane, a process that is calcium-dependent and associated with the loss of membrane asymmetry and disruption of the cellular cytoskeleton. Microvesicles carry membrane-derived receptors, proteins, lipids, carbohydrates, and genetic material, and their contents can affect the phenotype of recipient cells (Ståhl et al., 2019). Apoptotic bodies are the largest EVs, formed during the late stages of apoptosis, and can be several micrometers in size. Typically, apoptotic bodies are engulfed by phagocytes, such as macrophages, for final degradation, preventing the release of hazardous materials into the extracellular environment. Additionally, apoptotic bodies have been found to mediate intercellular communication, transferring biological signals and substances like proteins, metabolites, and nucleic acids to adjacent cells, which can regulate cellular function or signaling pathways to promote healing and tissue regeneration (Yu et al., 2023).

### 2.2 Biogenesis and cargo sorting of EVs

The biogenesis of EVs is a complex process that involves multiple cellular pathways and mechanisms. Exosomes are primarily derived from the endosomal compartment, specifically from multivesicular bodies (MVBs). The formation of exosomes begins with the inward budding of the endosomal membrane, which results in the creation of intraluminal vesicles (ILVs) within the MVBs. This process is facilitated by proteins known as tetraspanins, such as CD9 and CD63, which enrich the endosome membrane and contribute to the formation of ILVs. A key player in exosome biogenesis is the endosomal sorting complex required for transport (ESCRT), which includes four complexes (ESCRT-0, -I, -II, and -III) that recognize and sort ubiquitinated proteins into ILVs. The ESCRT machinery not only sorts cargo but also mediates membrane deformation and scission, leading to the release of ILVs as exosomes upon MVB fusion with the plasma membrane. In addition to the ESCRT-dependent pathway, there are ESCRT-independent pathways for exosome biogenesis. One such pathway involves the enzyme neutral sphingomyelinase-2 (nSMase2), which produces ceramide that can induce membrane curvature, facilitating ILV formation. Another pathway is mediated by the syndecan-syntenin-ALIX complex, which can bypass the early ESCRT machinery and directly influence exosome formation. Microvesicles, on the other hand, are formed through the outward budding and fission of the plasma membrane. This process can be triggered by various factors, including changes in lipid composition, actin-myosin contraction, and external signals such as calcium influx or hypoxia. The release of microvesicles is also regulated by proteins like ARF6, PLD, ERK, and MLCK, which initiate a cascade leading to vesicle shedding.

Recently, the advances in understanding the mechanisms of EVs cargo sorting have shed light on the complex processes that govern the packaging of biomolecules into EVs, which is crucial for their function in intercellular communication and as potential therapeutic agents. Proteomic and lipidomic analyses have revealed diversity in cargo composition among EVs from different cell types, highlighting distinct sorting mechanisms (Sherman et al., 2021). The heterogeneous nature of exosomes, irrespective of shared cellular origins, is explained in part by the coordination of ESCRT-dependent and -independent pathways, which may work together to sort cargo (Sherman et al., 2021). ESCRT is crucial for the sorting of ubiquitinated proteins into ILVs of MVBs, while ESCRT-independent pathways contribute to exosome biogenesis by the presence of tetraspanins CD81, CD9, and CD63 in exosomes (Lee et al., 2024). Proteins with posttranslational modifications like prenylation, myristoylation, ubiquitination, SUMOylation, and phosphorylation are also implicated in cargo sorting into exosomes (Ozkocak et al., 2022). Recently, a signal peptide sequence termed as the exosome-binding peptide (EBP) has been identified within the Wnt7a protein, which is crucial for the secretion of Wnt7a on EVs following muscle injury. Moreover, addition of EBP to an unrelated protein directed secretion on EVs, indicating the selective sorting of proteins into EVs in SKM (Gurriaran-Rodriguez et al., 2024). The RNA composition of exosomes varies depending on the cellular context, with RNA-binding proteins (RBPs) playing a pivotal role in the selective sorting of small RNAs, including miRNAs and tRNAs, into exosomes. For instance, miR-23a is a miRNA highly expressed in SKM. In the dexamethasone-induced muscle atrophy, exosomes encapsulate an increased amount of miR-23 without a change in their quantity, thereby reducing the decrease in miR-23 expression within the myotubes (Hudson et al., 2014). Whether the specific RBPs is involved in selective sorting of miR-23 into exosomes in SKM remains to be determined. Specific sequence motifs within cargo molecules are recognized by the sorting machinery, facilitating their packaging into EVs, with certain motifs like CGGGAG being strong examples for miRNAs. hnRNPA2B1, hnRNPK, SYNCRIP, and FMRP, have characterized motifs that contribute to the selective incorporation of associated RNAs into EVs. Additionally, modifications such as SUMOylation and phosphorylation, as well as LC3 conjugation, have been linked to the loading of small noncoding RNA species into EVs within the secretory autophagy pathway (Lee et al., 2024). Collectively, the selective sorting of cargo molecules into EVs is a critical determinant of EV heterogeneity during biogenesis (Lee et al., 2024).

### 2.3 Uptake mechanism of EVs

Most EVs do not seem to display tropism for a specific cell type; they are secreted by donor cells and target recipient cells non-selectively, and the uptake mechanism depends on the recipient cells (Horibe et al., 2018; Gabisonia et al., 2022). The cargos in EVs have been reported to target recipient cells through multiple mechanisms (Yue et al., 2020). The most straightforward mechanisms of EV uptake involves direct fusion of the EVs membrane with the plasma membrane of the target cell. This process releases the EVs cargo directly into the cytosol of the recipient cell and is thought to be facilitated by specific lipid compositions and membrane proteins on both the EVs and the target cell. EVs can be taken up into various specific recipient cells through internalizing mechanisms such as clathrin-, caveolin-, and lipid-raft-mediated endocytosis, and they subsequently merge into endosomes or are moved to lysosomes for degradation after being endocytosed (He et al., 2018; Yue et al., 2020). Cells like macrophages and dendritic cells can internalize EVs through phagocytosis, a process typically reserved for the internalization of large particles or pathogens. EVs are engulfed within a phagosome, which then fuses with lysosomes for degradation or processes the internalized cargo. EVs can also bind to specific receptors on the surface of recipient cells, triggering endocytosis through signaling pathways that lead to vesicle formation (O’Brien et al., 2022). These mechanisms can vary depending on the type of cell and its physiological state, as well as the specific cargo and surface markers present on the EVs.

## 3 SKM-EVs in SKM

SKM is a highly dynamic and plastic organ that requires complex regulatory circuits to maintain its biofunctional integrity. EVs have a substantial role in this regulation. They can be released and taken up by muscle fibers, indicating that SKM-EVs mediate the function and health of SKM itself, primarily with respect to muscle regeneration (Forterre et al., 2014a; Choi et al., 2016; Zanotti et al., 2018), repair, and metabolic regulation (Aoi and Tanimura, 2021; Mytidou et al., 2021).

### 3.1 SKM-EVs in muscle regeneration and repair

SKM-EVs may regulate myogenesis, muscle regeneration and injury repair through controlling the fate of muscle satellite cells (MSCs). Myogenesis begins with myoblast proliferation, followed by differentiation, which is regulated by a family of transcription factors (Hernández-Hernández et al., 2017). Pax7 is mainly expressed in quiescent MSCs, and MSCs are activated following muscle injury; they then begin to express either Myf5 or MyoD, followed by myogenin expression (Cornelison and Wold, 1997; Kumar et al., 2009). Activation of MSCs is a key element in this process, as they are located in close proximity to the muscle fibers and interact with the dynamic microenvironment (Dumont et al., 2015; Lehka and Rędowicz, 2020).

Myotube-secreted EVs can be taken up by myoblasts, inhibiting myoblast proliferation and promoting differentiation through downregulation of cyclin D1 and upregulation of myogenin (Forterre et al., 2014a). In injured muscle, MSCs treated with myoblast-derived EVs have been found to show upregulation of Pax7 and PPARγ; transcriptome and proteome analyses of these treated MSCs revealed the involvement of multiple biological processes, including proliferation and differentiation of MSCs, muscle regeneration, muscle atrophy, and inflammatory response to muscle injury (Ji et al., 2022). Similarly, stem cells treated with human myoblasts-derived EVs have been reported to fuse, resulting in a myotube-like phenotype with increased expression of myogenic proteins (Choi et al., 2016). The miR-206 family modulates optimal differentiation in skeletal myoblasts (Przanowska et al., 2020). EVs containing miR-206 are secreted by myogenic progenitor cells and ensure proper extracellular matrix deposition and activation of MSCs in response to hypertrophic stimuli (Fry et al., 2017). In the late stages of muscle repair, MSCs and myoblasts secrete EVs enriched in miR-206-3p and miR-27a/b-3p, which repress fibroadipogenic progenitor (FAP) adipogenesis, thereby enabling full muscle regeneration (Yu et al., 2024). C2C12-EVs from an inflammatory model, established *in vitro* by lipopolysaccharide stimulation, were observed to be endocytosed by both macrophages and myoblasts; they then induced M1 macrophage polarization and suppressed the M2 phenotype. This resulted in a sustained and aggravated inflammatory environment, thereby impairing myoblast differentiation and enhancing myogenic proliferation (Luo et al., 2021) (Table 1). C2C12-EVs from an inflammatory model treated with a cytokine mixture of TNF-α and INF-γ could activate molecular mechanisms contributing to muscle atrophy, including AMPK, p-38 MAPK and JNK, while inhibiting Akt-mediated myogenic signals (Kim et al., 2018). Duchenne muscular dystrophy fibroblast-derived EVs induced phenotypic conversion of normal fibroblasts to myofibroblasts thereby increasing the fibrotic response; this conversion was related to transfer of high levels of miR-199a-5p and reduction of its target caveolin-1 (Zanotti et al., 2018).

**TABLE 1:** Cargos in skeletal muscle derived extracellular vesicles play a role in various organs.

Target organ		Species	EVs source	EVs cargo	Recipient cell	Model/Disease	Result/Functions	References
SKM		Mice	C2C12 cells	NA	C2C12 cells	LPS treatment	Impaire myogenic differentiation and enhance proliferation	Luo et al. (2021)
		Human	Dystrophic fibroblast	miR-199a-5p	Normal fibroblasts	DMD	Conversion from normal fibroblasts to myofibroblasts	Zanotti et al. (2018)
		Mice	C2C12 cells	Palmitate	C2C12 cells	Palmitate treatment	Transfer the deleterious effect of palmitate on muscle cells	Aswad et al. (2014)
		Mice	C2C12 cells	NA	MSCs	BaCl2 induced injury	Promote the regeneration	Ji et al. (2022)
		Mice	Myotube	miR-133a	Myoblast	Normal C2C12 culture conditions	silenced Sirt1	Forterre et al., (2014a)
		Mice	Quadriceps, gastrocnemus	RAB35, intramuscular cholesterol contents	C2C12 cells, adipocytes	IR, obesity	Induce lipid storage in adipocytes and increase lipid uptake and fatty acid oxidation in muscle cells	Jalabert et al. (2021)
Bone		Mice	Atrophic SKM	NA	BMSCs	Disuse	Attenuate osteogenesis and enhance osteoclastogenesis	Huang et al. (2023)
		Mice	SKM tissue	lactate dehydrogenase A	BMSCs	Treadmill	Promote osteogenic differentiation of BMSCs	Ma et al. (2023)
		Mice	C2C12 cells	miR-92a-3p	BMSCs	Osteoporosis	Promote the proliferation and osteogenic differentiation of BMSCs	Xu et al. (2023)
		Mice	Myoblast	Prrx2	BMSCs	Osteoporosis	Promote osteogenic differentiation of BMSCs	Li et al. (2023)
		Mice	C2C12 cells	miR-34a	BMSCs	Aging	Induce BMSC senescence	Fulzele et al. (2019)
Cardiovascular system		Mice	SKM by remote ischemic conditioning	Musclin, CD71, VEGF, and b2-AR	Heart	Congestive heart failure	Alleviate heart failure	Homme et al. (2021)
		Rat	Plasma of trained mice	miR-342-5p	Cardiomyocytes	Swimming	Activate protective networks within the cardiovascular system	Hou et al. (2019)
		Mice	C2C12 cells	miR-130	Human umbilical vein endothelial cells	NA	Increase HUVEC ROS by activating the NF-κB pathway	Nie et al. (2019)
		Mice	Oxidative myofiber	nitric oxide synthase	Endothelial cells	NA	Promote endothelial cell migration and tube formation	Kargl et al. (2023)
Nervous system		Mice	C2C12 cells	interleukin-6, BDNF, and FDNC5	Primary hippocampal neurons	Engineered neuromuscular tissue model	Promotes the innervatation of SKM by elevating expression of mRNAs encoding neurotrophic myokines	Huang et al. (2024a)
		Mice	gastrocnemius, Plantaris, EDL, soleus	miR-133a	NIH 3T3 cells	Denervation	Downregulate the protein expression of Smarcd1 and Runx2	De Gasperi et al. (2017)
		Mice	C2C12 cells	miR-29b-3p	neuronal SH-SY5Y cells	Aging	Downregulated neuronal-related genes and inhibited neuronal differentiation	Yang et al. (2020a)
		Human	I muscle fibers in human	pro-BDNF	NA	Resistance exercise	Exercise with higher lactate augmented levels of plasma mature BDNF	Edman et al. (2024)
		Rat	SKM after exercise	miR-484	Neurons	Ferroptosis	Suppress Acsl4 expression to inhibit ferroptosis	Huang et al. (2024b)
		Rat	MDSCs	NA	Schwann cells, dorsal root ganglion cells	Ferroptosis	Suppress cell ferroptosis, enhanced viability in Schwann cells and dorsal root ganglion cells	Liu et al. (2024)
Adipose tissue		Pig	MSCs	miR-146a-5p, miR-221-5p	Porcine pre-adipocytes	NA	Involved in lipid-related metabolism processes probably	Li et al. (2021a)
		Mice	SKM	miR-146a-5p	Pre-Adipocytes	HFD	Mediate adipogenesis and fatty acid absorption	Qin et al. (2023)
		Mice	MSCs and their derived myoblasts	miR-206-3p, miR-27a/b-3p	FAPs	LPS treatment	Repress FAP adipogenesis, enabling full muscle regeneration	Yu et al. (2024)
Kidney		Mice	TA	AAV-miR-23a/27a	Kidney; SKM	Diabetes	Prevent diabetes-induced muscle cachexia, attenuate renal fibrosis	Zhang et al. (2018)
		Mice	TA	AAV-miR-29a	Kidney; SKM	UUO	Improve renal fibrosis in UUO mice	Wang et al. (2020)
		Mice	TA	Lamp2b-RVG vector; Exo/miR29	Kidney; SKM	UUO	Attenuate muscle loss and renal fibrosis	Wang et al. (2019b)
		Mice	TA	pLamp2b/MSTP-miR-26a	Kidney; heart	Uremic cardiomyopathy	Mitigated uremic cardiomyopathy in CKD mice	Wang et al. (2019a)
		Mice	HEK293 cells; TA	Exo/miR-26a	Kidney	UUO	Suppress muscle atrophy and renal fibrosis in CKD	Zhang et al. (2019)
Other organs	Pancreas	Mice	Quadriceps muscle	NA; miR-16	MIN6B1 cells; beta cell	IR	Induced proliferation of MIN6B1 cells and isolate islets; regulate Ptch1	Jalabert et al. (2016)
	Liver	Mice	Plasma of trained mice	miR-133a, miR-133b	3T3-L1 cells; primary hepatocytes	HIIT	Decrease expression of FoxO1 in the livers	Castaño et al. (2020)
	Macrophages	Mice	C2C12 cells	miR-206-3p, miR-378a-3p, miR-30d-5p, and miR-21a-5p	bone marrow-derived macrophages	LPS treatment	Suppress macrophage inflammatory responses	Yamaguchi et al. (2023)
	Lung	Human	Quadriceps muscle, plasma	miR-1, miR-2-6,and miR-499; miR-206	NA	COPD	educed expression of myomiRs in quadriceps muscle in COPD patients	Donaldson et al. (2013)

NA, not applicable; LPS, lipopolysaccharide; DMD, Duchenne muscular dystrophy; MSCs, muscle satellite cells; SKM, skeletal muscle; BMSCs, bone marrow mesenchymal stem cells; BDNF, brain-derived neurotrophic factor; FAPs, fibroadipogenic progenitors; UUO, unilateral ureteral obstruction; TA, tibialis anterior; MDSCs, muscle-derived stem cells; IR, insulin resistance; HIIT, high-intensity interval training; COPD, chronic obstructive pulmonary disease.

### 3.2 SKM-EVs in muscle metabolic regulation

EVs from palmitate-treated C2C12 cells have been reported to be enriched in palmitate, suggesting that they are likely to transfer the deleterious effect of palm oil between muscle cells by transferring lipids (Aswad et al., 2014).

Myotube-derived exosomal miR-133a was found to silence Sirt1, a gene involved in muscle proliferation, mitochondrial biogenesis and fatty acid oxidation, suggesting that myotube-EVs potentially regulate muscle metabolic homeostasis and SKM growth (Forterre et al., 2014b). Moreover, EVs released by atrophic insulin-resistan (IR) OB-SKM (leptin-deficient) mice showed decreased RAB35 and increased intramuscular cholesterol contents, inducing lipid storage in recipient adipocytes and increased lipid uptake and fatty acid oxidation in recipient muscle cells, suggesting that crosstalk occurrs in muscle between muscle cells and adipocytes (Jalabert et al., 2021).

## 4 EVs in crosstalk between muscle and other organs

### 4.1 EVs in muscle–bone crosstalk

Owing to their anatomical connection, muscle and bone inevitably interact with each other functionally. This functionally interaction is primarily reflected in the translation of mechanical strain into biochemical responses and endocrine effects mediated by EVs (Herrmann et al., 2020).

SKM-EVs can travel through the bloodstream to reach bone marrow mesenchymal stem cells (BMSCs); thus, they can ameliorate bone-related diseases through rebalancing bone formation and resorption (Xu et al., 2018; Chen et al., 2024). BMSCs contribute to bone regeneration through self-replication and differentiation into various cell types, and through their essential roles in fracture repair, bone development, and overall bone health (Wang et al., 2013). *In vitro* studies have demonstrated that EVs from normal SKM can be phagocytized by BMSCs and osteoclasts (OCs), promoting osteogenic differentiation of BMSCs while inhibiting OC formation (Huang et al., 2023). By contrast, EVs from atrophic SKM attenuated osteogenesis of BMSCs and strengthened osteoclastogenesis of monocytes (Huang et al., 2023). *In vivo* experiments have shown that SKM-EVs from normal mice can efficiently reverse disuse osteoporosis by enhancing bone formation and suppressing bone resorption (Huang et al., 2023). Furthermore, SKM-EVs can deliver glycolytic enzymes such as lactate dehydrogenase A into BMSCs to enhance glycolysis, thereby regulating bone resorption mediated by OCs and promoting osteogenic differentiation of BMSCs (Ma et al., 2023).

In addition, EVs containing miR-92a-3p, derived from mechanical stress-induced myoblasts, promote the proliferation and osteogenic differentiation of BMSCs via the miR-92a-3p/PTEN/AKT signaling pathway and have exhibited therapeutic effects on glucocorticoid-induced osteoporosis in mice *in vivo* (Xu et al., 2023). Likewise, myoblast-derived exosomal Prrx2 contributes to transcriptional activation of MIR22HG, activating the YAP pathway by sponging miR-128, thereby facilitating osteogenic differentiation of BMSCs (Li et al., 2023). C2C12-EVs have been found to show a significant increase in miR-34a with age, and these EVs decreased the viability of BMSCs and increased BMSC senescence. EVs from C2C12 cells overexpressing miR-34a were also observed to translocate to bone, inducing senescence and downregulating Sirt1 expression in primary bone marrow cells (Fulzele et al., 2019).

Other studies have also confirmed that musculoskeletal interaction is bidirectional. For instance, BMSC-EVs mitigated the reduction in myotube diameter induced by dexamethasone, and further mechanistic analysis revealed that the expression of miR-486-5p in C2C12 cells was upregulated when FoxO1 was downregulated following treatment with BMSC-EVs. These results indicate that BMSC-derived EVs inhibit dexamethasone-induced muscle atrophy via the miR486-5p/Foxo1 axis (Li Z. et al., 2021). Moreover, miR-145-5p, which is relevant to muscle regeneration, has been found in high concentrations within EVs from human fetal cartilage-derived progenitor cells. These EVs effectively induced the expression of muscle markers (MyoD, MyoG, and MyHC) and myotube formation, and significantly increased muscle fiber cross-sectional area and muscle mass in a rat model of sarcopenia (Shin et al., 2024). Senescent BMSC-EVs impaired myogenesis of MSCs; this effect was attributed to the CD81 on the surface of BMSC-EVs binding to membrane proteins of MSCs, which caused fewer formation of myotubes and lower expression of MyoD, MyoG, and MyHC (Dai et al., 2022).

### 4.2 EVs in muscle–cardiovascular system crosstalk

Communications between SKM and the heart and/or blood vessels have been extensively studied, given that various diseases are known to involve both SKM and the cardiovascular system (Murach and McCarthy, 2017). For instance, aging adults with sarcopenia may experience alterations in myocardial structure and function, leading to a syndrome known as “cardio-sarcopenia” (Keng et al., 2019). Abnormalities in SKM, including morphologic, histologic, and enzymatic changes, as well as metabolic and autonomic dysfunction, have also been observed in patients with chronic heart failure (Georgiadou and Adamopoulos, 2012).

Su et al. (2021) reported that transplantation with EVs from an allogeneic myogenic progenitor (MPC-EVs) improved cardiac function in mice with X-linked muscular dystrophy (mdx) mice; treatment with MPC-EVs for 2 days increased Bcl-2 and decreased IL-6 protein in mdx mouse hearts, suggesting that anti-apoptotic and anti-inflammatory effects might be responsible for the improvement in heart function associated with MPC-EVs. In a similar model, a beneficial increase in dystrophin expression following cardiosphere-derived EVs treatment was observed in mdx mice; this was possibly due to the transfer of miR-148a to SKM cells (Aminzadeh et al., 2018). The results of another study indicate that remote limb ischemic conditioning triggers the release of SKM-EVs containing musclin, CD71, VEGF, and b2-adrenergic receptor (b2-AR), thereby alleviating heart failure in mice (Homme et al., 2021).

There is a consensus based on previous research that exercise is an effective measure for preventing and treating cardiovascular diseases, with SKM being the primary organ involved. In fact, exercise stimulates the release of EVs enriched with irisin, miR-486-5p, and miR-342-5p from SKM; this result in the activation of protective networks within the cardiovascular system, including the AMPK/PI3K/AKT pathway, transforming growth factor-beta 1 (TGFβ1)/Smad2/3 pathway, and PI3K/AKT/VEGF pathway (Hou et al., 2019; Wang et al., 2024).

Murach and McCarthy (2017) proposed a panel of miRNAs that are altered in damaged human cardiac tissue and could serve as an preliminary template for heart–SKM communication during heart failure; this comprised miR-1, miR-21, miR-24, miR-29b, miR-133a and b, miR-199, miR-208, miR-214, and miR-499, which are consistently affected in human heart failure, regardless of its origin and severity.

In terms of vascular health, SKM fibers regulate surrounding endothelial cells by secreting numerous angiogenic factors, some of which are encapsulated in EVs. Nie et al. demonstrated that C2C12-EVs enhance angiogenesis and endothelial cell functions. They attributed this effect to the transfer of miR-130 to human umbilical vein endothelial cells (HUVECs), which increases HUVEC reactive oxygen species and activates the NF-κB pathway (Nie et al., 2019). Muscle fibers are broadly classified as oxidative (OXI) or glycolytic (GLY) depending on their 223metabolic characteristics. Following the observation that OXI muscle fibers secreted more EVs than GLY muscle tissue, endothelial cells were treated with OXI-EVs and GLY-EVs to investigate their angiogenic signaling potential. The OXI-EVs promoted greater endothelial cell migration and tube formation compared with GLY-EVs, and this effect may have been mediated through nitric oxide synthase-related pathways (Kargl et al., 2023).

### 4.3 EVs in muscle–nervous system crosstalk

Neuronal innervation and firing have important roles in regulating the secretory activities of SKM. A study using an engineered neuromuscular tissue model, comprising SKM innervated by motor neurons, showed that innervated SKM exhibited elevated expression of mRNAs encoding neurotrophic myokines, including interleukin-6, BDNF, and FDNC5 (Huang K.-Y. et al., 2024). Upon glutamate stimulation, innervated muscles were found to secrete higher levels of irisin and EVs containing a more diverse array of neurotrophic miRNAs compared with neuron-free muscles (Huang K.-Y. et al., 2024). By contrast, denervation resulting from nerve injury, induces rapid SKM fiber degeneration through the activation of atrophy-related signaling and subsequent disassembly of sarcomeres, leading to significant changes in the miRNA profile of SKM-EVs and altering normal muscle function (Rodríguez and Cabello-Verrugio, 2024). Specifically, miR-206 levels increase in denervated SKM, whereas miR-1, miR-133a, and miR-133b levels decrease. EVs from denervated myofibers can downregulate the protein expression of Smarcd1 and Runx2 in recipient cells, such as NIH 3T3 cells, via the action of miR-133a (De Gasperi et al., 2017) *In vivo* experiments h ave shown that EVs obtained from denervated muscles can influence the accuracy of regeneration in a femoral nerve model (Madison and Robinson, 2019). Moreover, miR-29b-3p has been shown to play a key part in muscle atrophy, being upregulated in normal and premature aging mouse muscle and plasma and in the blood of aging individuals; circulating levels of miR-29b-3p were also negatively correlated with relative appendicular SKM mass (Yang C.-P. et al., 2020). C2C12-derived EVs containing miR-29b-3p were taken up by neuronal SH-SY5Y cells, leading to downregulation of neuronal-related genes and inhibition of neuronal differentiation (Yang C.-P. et al., 2020).

According to the recent studies, exercise and the secretome of SKM can improve a broad range of brain functions related to vascularization, neuroplasticity, memory, sleep, and mood (Delezie and Handschin, 2018). BDNF is among the biological factors secreted by contracting SKM in response to exercise training, promoting brain plasticity (Jaberi and Fahnestock, 2023). Edman et al. (2024) showed that pro-BDNF is highly expressed in human SKM, particularly in type I fibers, with expression increasing after exercise. Moreover, exercise with higher lactate augmented levels of plasma mature BDNF, which were partly derived from the release and cleavage of pro-BDNF in SKM and partly from neural and other tissues. However, exercise negatively affects individuals with myalgic encephalomyelitis/chronic fatigue syndrome (ME/CFS), who suffer from exercise intolerance (Bateman et al., 2021). The exosomal proteome in ME/CFS patients differs from that in healthy individuals and is involved in numerous pathways and systems, including coagulation processes, muscle contraction (both smooth muscle and SKM), cytoskeletal proteins, the immune system, and brain signaling. Changes in Exo proteins caused by exercise have been shown to be strongly correlated with symptom severity in ME/CFS (Giloteaux et al., 2024).

Ferroptosis is the most dominant form of programmed cell death in neurons. Huang et al. reported that preconditioning exercise prior to stroke improved neurological function and decreased the infarct area in rats with ischemic stroke; this was owing to exercise-induced SKM-EVs being enriched in miR-484, which suppresses Acsl4 expression to inhibit ferroptosis (Huang M. et al., 2024). Analogously, muscle-derived stem cells (MDSCs), a diverse group of multipotent cells, hold promise for peripheral nerve regeneration, and the EVs from MDSCs have been shown to effectively suppress cell ferroptosis and enhance viability in Schwann cells and dorsal root ganglion cells (Liu et al., 2024). Moreover rats treated with MDSC-EVs exhibited improvements in sciatic nerve function caused by increases expression of BDNF and P62 and decreases in expresion of Keap1, Nrf2, and HO-1 in Schwann cells (Liu et al., 2024).

### 4.4 EVs in muscle–adipose tissue crosstalk

Adipose tissue (AT) is an active endocrine organ that produces various hormones and cytokines to regulate appetite, metabolism, inflammation, and immune responses (Huang and Xu, 2021). For example, adiponectin, which is mainly secreted by adipocytes, enhances insulin sensitivity and regulates glucose metabolism (Diamond and Eichler, 2002). A SKM and AT communicate with each other via EVs loaded with myokines, adipokines, and miRNAs, thereby maintaining overall health (Guo et al., 2023).

Differential expression of miRNAs between MSCs-EVs and adipose EVs has been reported, with miR-146a-5p and miR-221-5p upregulated in MSCs-EVs; KEGG analysis showed that these miRNAs were mainly involved in lipid-related metabolism processes probably through multiple signaling pathways (Li W. et al., 2021). Specifically, miR-146a-5p functions as a negative regulator of the PPARγ signaling pathway by directly targeting the GDF5 gene to mediate adipogenesis and fatty acid absorption (Qin et al., 2023). It can also suppress intramuscular preadipocytes proliferation by directly targeting SMAD4 to attenuate TGF-β signaling, as well as inhibiting differentiation of intramuscular preadipocytes by targeting TRAF6 to weaken the downstream AKT/mTORC1 signaling pathway (Zhang et al., 2021). These findings demonstrate that SKM-EVs enriched in miR-146a-5p can inhibit adipogenesis of pre-adipocytes.

Fibroadipogenic progenitors (FAPs) are the main source of intramuscular adipose tissue (IMAT); muscles without FAPs exhibit decreased IMAT infiltration but also deficient muscle regeneration, indicating the importance of FAPs in the repair process. After acute muscle injury, FAPs are activated and release EVs enriched with miR-127-3p, which targets the sphingosine-1-phosphate receptor S1pr3 and activates myogenesis (Yu et al., 2024). Indeed, intramuscular injection of EVs from FAPs have been shown to accelerate regeneration of injured muscle. Notably, SKM-EVs can negatively affect FAP adipogenic differentiation during the muscle regeneration process, with little effect on FAP cell proliferation. In the late stages of muscle repair, MSCs and their derived myoblasts secrete SKM-EVs enriched in miR-206-3p and miR-27a/b-3p, which repress FAP adipogenesis, enabling full muscle regeneration (Yu et al., 2024). This reciprocal communication between FAPs and muscle cells via miRNAs in their secreted EVs has a critical role, limiting IMA infiltration while stimulating muscle regeneration. This represents an important mechanism for SKM repair and homeostasis.

FAPs also have the capacity to differentiate into fibrogenic, white, and beige adipose tissue (BAT), and FAPs that have assumed a BAT differentiation state (FAP-BAT) have proven to be efficacious in treating muscle degeneration in numerous injury models. Davies et al. used EVs from FAP-BAT cells (Exo-FB) to treat C2C12 cells and mouse embryonic fibroblasts *in vitro*; they observed that Exo-FB significantly promoted myotube fusion in C2C12 cells and induced differentiation into myotubes in mouse embryonic fibroblasts. Mice treated with Exo-FB at the time of rotator cuff injury displayed markedly reduced muscle atrophy and fatty infiltration (Davies et al., 2022). However, in a muscle disuse atrophy model, activation of IL-1β related to disuse was shown to directly affect miRNA cargos in FAP-derived EVs; these included miR-let-7c, miR-let-7b, miR-181a, and miR-124, which have previously been demonstrated to have important roles in cellular senescence and muscle atrophy (Parker et al., 2022).

EVs from perimuscular adipose tissue (PMAT) were transplanted into hindlimb thigh muscles of young mice, where they resulted in a reduction in integrin α7/CD29-double positive MSCs. In addition, EVs from the PMAT were enriched in Let-7d-3p miRNA, which targets HMGA2 to inhibit proliferation of MSCs. Activation of NF-κB and reduction of the Lin28 A/B, Let-7 miRNA repressor led to the accumulation of Let-7d-3p in EVs of aged PMAT, indicating that AT-derived miRNAs could have a key role in sarcopenia (Itokazu et al., 2022).

In the context of circadian rhythm disruption induced by Bmal1 interference, exosomal miR-22-3p derived from adipocytes was found to taken up by SKM cells, leading to the IR *in vitro*. Moreover, miR-22-3p in the circulation was positively correlated with clinical IR-associated factors, indicating that exosomal miR-22-3p in the circulation could serve as biomarker for SKM IR (Zhang et al., 2023). EVs derived from adipocytes of high fat diet (HFD) fed mice were also found to be enriched in miR-27a, which could induce IR in C2C12 cells by inhibiting PPARγ and its downstream genes (Yu et al., 2018). In addition, triglyceride accumulation increased and glucose uptake decreased in C2C12 cells treated with adipocyte-EVs from HFD-fed obese mice, demonstrating that these EVs contributed to the development of IR in C2C12 cells (Kim et al., 2022).

### 4.5 EVs in muscle–kidney crosstalk

Chronic diseases such as chronic kidney disease and diabetes are frequently accompanied by muscle wasting and renal fibrosis, which significantly contribute to the morbidity and mortality associated with these conditions. Recent research has shown that EVs have a role in mediating the pathogenesis of these complications and has also highlighted the therapeutic potential of exosomal miRNAs as delivery vehicles.

Notably, levels of miR-23a and miR-27a were found to be decreased in the SKM of mice with chronic kidney disease induced by streptozotocin. Then, an adeno-associated virus (AAV) carrying the precursor RNA of miR-23a/27a/24-2 was injected into the tibialis anterior muscle of diabetic mice (Zhang et al., 2018). This led to increased levels of miR-23a and miR-27a in the SKM. Moreover, treatment with AAV-miR-23a/27a attenuated the diabetes-induced reduction in muscle cross-sectional area and improved muscle function. Levels of miR-23a and miR-27a in serum EVs and kidney were significantly increased compared with those in control-virus-injected mice following intramuscular injection of AAV-miR-23a/27a. These data support the possibility that the muscle may participate in crosstalk with kidney by transfer of miRNAs via EVs. Another study by Wang et al. supported this possibility: in a mouse model of unilateral ureteral obstruction (UUO), miR-29 levels were decreased in SKM and kidneys, consistent with the increased muscle atrophy and renal fibrosis observed in this model (Wang et al., 2020). When a recombinant AAV carrying miR-29a (AAV-miR-29a) was injected into the tibialis anterior muscle of mice to overexpress miR-29a, both muscle atrophy and renal fibrosis were mitigated, indicating crosstalk between muscle and kidney (Wang et al., 2020). Similarly, intramuscular injection of Exo-encapsulated miR-29 (Exo/miR-29) increased muscle cross-sectional area and decreased expression of atrophy markers such as TRIM63/MuRF1 and FBXO32/atrogin-1. This Exo/miR-29a was transferred from the site of injection in the muscle to other organs, including the kidneys, where it was absorbed. The presence of Exo/miR-29a was found to partially alleviate renal fibrosis in UUO mice, as indicated by diminished levels of TGF-β, a-SMA, fibronectin, and collagen 1A1 within kidney tissue (Wang H. et al., 2019). Similarly, miR-26a levels were decreased in serum EVs of UUO mice, and miR-26a overexpression induced by injection of Exo/miR-26a into SKM of UUO mice increased muscle weight and attenuated muscle atrophy (Wang B. et al., 2019). Furthermore, Exo/miR-26a was absorbed by the kidney and limited renal fibrosis by directly suppressing connective tissue growth factor and TGF-β1, which are key profibrotic proteins (Zhang et al., 2019).

### 4.6 SKM-EVs in other metabolic diseases and immunological crosstalk

SKM-EVs taken up by pancreatic beta cells have a role in the etiology of diabetes. Quadriceps muscle derived EVs were purified from mice fed with a standard chow diet (SCD) fed or a standard diet enriched with 20% palmitate (HPD) and injected into mice. *In vivo*, muscle EVs were found to be taken up by the pancreas 24 h post-injection. *In vitro*, when beta cells were treated SCD-EVs and HPD-EVs, both types of Exo transferred proteins and miRNAs to MIN6B1 cells and modulated gene expressions. However, only HPD-ELVs induced proliferation of MIN6B1 cells and isolated islets; in addition, miR-16, which is overexpressed in HPD-EVs, was transferred to MIN6B1 cells and regulated Ptch1, which is involved in pancreas development. These findings suggest that the effects of palm oil on islet size observed *in vivo* were reproduced *in vitro* by under treatment with HPD-EVs (Jalabert et al., 2016). In another study in mice, pancreatic cancer-derived EVs were found to induce SKM IR; miRNA-let-7b-5p, which was identified as the key exosomal miRNA, promoted IR in C2C12 cells, which led to increased lipid accumulation, activation of the STAT3/FOXO1 axis, and decreased expression of IRS-1 and GLUT4. Moreover, the target gene of let-7b-5p (RNF20, which encodes an E3 ubiquitin ligase) was found to improve C2C12 IR by downregulating STAT3 protein expression via ubiquitination-mediated protein degradation (Wang et al., 2023). In a type 2 diabetes model, levels of miRNAs including miR133a, miR-206,and miR-16 were markedly changed in SKM; these changes affected beta cell survival and function and improve glucose uptake and systemic metabolism through a mechanism mediated by EVs (Barlow and Solomon, 2018).

High-intensity interval training (HIIT) modifies the miRNA profiles of circulating EVs in mice, including significant increases in miR-133a and miR-133b. Treatment of sedentary mice with EVs isolated from the plasma of trained mice has been shown to improve glucose tolerance and insulin sensitivity, and to decrease plasma levels of triglycerides. MiR-133a and miR-133b were upregulated by HIIT and targeted insulin-regulated transcription factor FoxO1, resulting in decreased expression of FoxO1 in the livers of trained and Exo-treated mice (Castaño et al., 2020). These findings indicate that circulating EVs released by SKM carry a specific miRNA signature that is modified by exercise and induce expression changes in the liver that affect the metabolic profile of the whole body. Analyses using lipidomics have revealed that SKM-EVs can transfer lipids among muscle cells. Furthermore, under condition of palmitate-induced IR, secretion of SKM-EVs enriched with palmitate is enhanced (Aswad et al., 2014).

Myotube-EVs are thought to increase the production of itaconate via upregulation of IRG1 expression and exhibit an anti-inflammatory effect in macrophages. This anti-inflammatory effect could be attributed to miR-206-3p, miR-378a-3p, miR-30d-5p, and miR-21a-5p in myotube-EVs and their involvement in the PI3K-Akt and JAK-STAT pathways (Yamaguchi et al., 2023).

Levels of muscle-specific miRNAs, including miR-1, miR-2-6,and miR-499, have been reported to be reduced in quadriceps muscle and increased in plasma of patients with chronic obstructive pulmonary disease (COPD), who often have a sarcopenic condition. In plasma samples from 103 chronic obstructive pulmonary disease patients and healthy controls, plasma miR-499 and miR-206 levels were associated with inflammatory responses in SKM and the circulation (Donaldson et al., 2013).

## 5 Challenges and future direction

Despite the immense potential of SKM-EVs in basic research and clinical applications, there are still challenges to overcome. One challenge is the variability in the purity and heterogeneity of EVs isolated using different methods, which can substantially impact the outcomes and the comparability of results across various studies. Different techniques such as ultracentrifugation, density ultracentrifugation, size exclusion, and immuno-affinity can yield EVs with different structural and biomolecular characteristics (Tang et al., 2017; Patel et al., 2019; Chen et al., 2021). For instance, ultracentrifugation can yield high-purity EV preparations but may result in the loss of some EVs due to pelleting artifacts or contamination with other cellular debris. Size exclusion chromatography is a gentle method that minimizes EV aggregation and degradation. However, it may also result in the co-elution of proteins and other macromolecules, which can affect the purity of the EV preparation. Methods like ExoQuick or Total Exosome Isolation Reagent use polymers to precipitate EVs out of the solution. These methods are relatively quick and cost-effective but may introduce variability in EV yield and purity. They can also co-precipitate proteins and other macromolecules, leading to potential contamination. The labeling of EVs for tracking purposes presents another set of challenge (Brealey et al., 2024). Both endogenous and exogenous labeling methods, such as the use of lipophilic dyes or protein-conjugated dyes, can lead to variations in the EVs’ behavior and potential for free dye leakage, which may affect the accuracy of uptake and trafficking studies. The choice of fluorescent probe can also impact the study outcomes, as different probes may have varying affinities for EVs and different impacts on EV integrity. In addition, the method of EV labeling can influence the detection and analysis of EV interactions with recipient cells. Some fluorescence signals might originate from dye transfer rather than actual uptake or content delivery of EVs, which can lead to misinterpretation of experimental results (Brealey et al., 2024). Therefore, researchers must consider the specific requirements of their experiments to ensure the high yield, purity, or specific subclasses of EVs and there is a need for a global consensus on standardization to ensure the reproducibility and reliability of EV studies.

In summary, EVs are nanoscale extracellular vesicles that carry a variety of bioactive molecules, which can be transported over long distances in body fluids and facilitate information exchange and functional regulation. Here, we have primarily discussed the pivotal regulatory role of SKM-EVs in mediating bio-information exchange between SKM and other organs. The cargos and circulating levels of SKM-EVs change in response to physiological and pathological stimuli, which in turn affect the pathophysiological fates of SKM. Thus, engineered EVs with modified contents and tissue-specific targeting ligands hold tremendous promise for use in targeted therapies to treat SKM-related diseases. Future research should further elucidate the intercellular communication signaling pathways mediated by EVs, as well as the key bioactive components within EVs and their mechanisms of action. Evaluation of the safety and efficacy of EVs as therapeutic tools will also be essential to promote clinical translation. Potential future directions include developing personalized diagnostic and treatment methods based on EVs, as well as systems for disease diagnosis and prognostics assessment.
